# Traction, as Applied to Tubercular Joint Conditions1Read at a meeting of the Buffalo Academy of Medicine, Nov. 8, 1905

**Published:** 1905-12

**Authors:** Prescott Le Breton

**Affiliations:** Buffalo, N. Y.


### BUFFALO MEDICAL JOURNAL.

VOL. LXI. DECEMBER, 1905. NO. 5

### ORIGINAL COMMUNICATIONS.

### Traction, as Applied to Tubercular Joint Conditions[1]

BY PRESCOTT LE BRETON, M. D., of Buffalo, N. Y.

THE value of traction, as applied for tubercular joint conditions, is not disputed, but its limitations, as to the length of time it should be used, has long been discussed. It is especially with reference to the hip joint that authorities are not agreed. A short review of the indications of traction outside the hip joint, where its place is well defined, is first in order.

At the wrist and elbow fixation by plaster of paris and the use of a sling are provided, and traction is not of value. At the shoulder, a shoulder-cap and sling are sufficient, and the weight of the arm provides enough pull to give comfort. At the ankle, fixation by plaster and the use of a Thomas knee brace which avoids weight bearing on the foot, are the routine measures. In acute tubercular troubles at the knee, where there is muscular spasm and some flexion, traction is employed until the acute symptoms have abated, and then fixation and protection by a Thomas knee brace are provided. The Thomas caliper brace prevents the heel from striking the ground, i. e., affords stilting. The other form of Thomas brace, in common use, by its extended bars, permits traction by adhesive straps, if muscular spasm has not disappeared.

In Pott's disease, traction is often of great service in different ways. The Bradford gas-pipe frame, for example, can be made to counteract spasm by bending back the bars opposite the site of the disease, thus hyperextending the spine. With disease in the cervical or upper dorsal spine, pain is relieved by traction on the chin and occiput, and for compression myelitis this extension is important. The inefficient jury-mast is now discarded for more elegant and serviceable supports. Occasionally, when the trouble is located in the lumbar region, the

1 Read at a meeting of the Buffalo Academy of Medicine, Nov. 8, 1905

psoas muscle is contracted and flexion of the thigh is steadily in evidence. In such an event traction in the line of the deformity by weight and pulley will cause the symptom to subside. When the tubercular trouble is limited to these portions of the body, however, it is evident that traction occupies but a minor part of the treatment, while prolonged fixation and protection are required.

Among those writers who, of late years, have discussed the place that traction occupies in the treatment of hip disease, Lorenz (*Journal of the American Medical Association,* Feb. 4, 1905) has been very outspoken. He believes that traction is employed far too long by the Americans and many Europeans. He has not used it for ten years. Untreated cases, cured by nature, show good results except for the deformity, and yet they have continued to use the limb and develop the muscles and other tissues. He believes that traction is a good agent to relieve pain at the outset, but that its prolonged use allows the leg to atrophy, the ligaments of the knee-joint to become lax, the bones to get brittle, while the disease is not shortened nor is deformity less likely to appear later on. He finds the thigh sensitive to motion at all times, but not to weightbearing, so he provides fixation throughout but allows the patient to bear the weight on the foot as soon as acute symtoms have disappeared. Plaster of paris casts furnish fixation during the first year, after which time a leather spica is substituted. The knee joint is not included in the plaster or leather, so that it is normally developed. Late in the course of the disease massage and exercises of the thigh are given, the motions being in the direction of extension and abduction to counteract the usual deformities of flexion and adduction.

Whitman, (Orthopedic Surgery), among American surgeons, who have not been perfectly satisfied with the ordinary traction walking brace, has attempted to modify this brace. Finding that the brace really afforded neither traction nor fixation to any satisfactory degree, he added first under the apparatus, a plaster spica. As this was heavy he next substituted a Thomas hip splint underneath. Then he made a combination of the two, by adding to the original Taylor brace a lateral thoracic bar and a chest band. The great object of these additions, of course, was to provide a fixation of the joint. He found his last modification satisfactory, as it immobilized the hip and knee joints, and partially fixed the lower spine. For acute phases of the disease with muscular spasm, recumbency and traction are employed by him. He discusses at length the relative merits of the American plan of providing traction by a Taylor brace, of the English method by the Thomas splint, and what may be called the German preference for a series of plaster spicas.

John Dane (*New York Medical Journal*, August 24, 1901), declares that after a trial of nearly two years he had to remove all the thoracic extensions, such as Whitman described, and has since confined himself to such modifications as would secure a more accurate and firm grasp of the pelvis. Believing that greater fixation was indicated, he describes a splint like the Taylor brace in which the pelvic arm is broadened, embracing the pelvis firmly, and allowing only about six degrees of motion at the joint instead of the thirty-five degrees allowed by the Taylor brace.

Lovett, in the same journal, suggests another combination. He believes that traction is superior to fixation, but at the same time believes that greater fixation is essential. In other words, that the joint should be limited as to motion well inside the limit set by Nature and shown by the muscular spasm. He also devised some years ago a combination of the Taylor and Thomas splints, but found that a thoracic bar could not be firmly applied to the chest. His device is to apply a leather spica to the chest and thigh, and over this to fasten a skeleton traction splint. Patients are not allowed to remove it oftener than once a week. His conclusions are that cases permitting not over twenty-five degrees of motion in flexion should be supplied with his modification. Cases allowing twenty-five to forty-five degrees may be treated by Dane's splint with high shoe and crutches; and further, that the use of the Taylor long traction splint should be limited to cases allowing well over forty-five degrees, with the addition of high shoe and crutches.

Packard (*American Journal of Orthopedic Surgery*, Vol. 1, p. 153), has discussed the question of the treatment of hip disease not as regards traction, but as regards the question of how long should treatment be continued without allowing some functional use of the affected member. He cited the case of a girl of fourteen, who came under his observation after recovery from double coxitis. He found that one leg, which she had constantly used, on account of its good position, directly after the subsidence of the acute symptoms, was a strong well-developed member, while the other, which had been practically at rest from its fixed position and deprived of functional use, was atrophied, short and weak. He says, "When we come to consider the likelihood of retarded growth and the weakness that is likely to follow a long-continued lack of function, would it not be more rational to substitute massage of the muscles and a protracted use of the joint after the subsidence of the active symptoms?" The discussion that followed at the meeting of the Orthopedic Association brought out the fact that the majority of the members were against such an innovation as weight-bearing and motion.

Enough has been quoted from the literature to show that the treatment of hip disease is not in a settled condition. It would seem best to decide the question of the form of treatment indicated according to the individual case, and according to the dictation of experience. It is well known, for example, that while the majority of acute cases are quickly relieved by rest and traction, there are a few which continue to grow worse, and who develop increased pain, night-cries, etc., until an incision into the joint is made, and the process opened up. There must be some grains of truth in the various policies advocated, and there is a correct time to apply each one as conditions present themselves. As men grow in experience, they can detect that in an individual case, fixation has not been sufficient, and that deformity has resulted. That in another case, traction has been used too long, and the knee is lax and weak. That in still another case, weightbearing was allowed too soon, and a relapse with abscess has been due, apparently, to this fact. The writer, during the past four years, has tried to follow a combination plan of treatment, which has given good results. Reliance has been placed mostly on fixation in a good position, and traction has been employed while spasm, and sensitiveness are present. The patient is kept in bed, with fixation and traction applied for a period of about six weeks until it is found that the joint is no longer sensitive to a light blow over the trochanter or the heel, and until the muscle spasm is greatly abated. Then the patient is supplied with high shoe and crutches, and is allowed to get about wearing a plaster spica from the nipple line down to the lower calf. If the case is mild in type, a leather spica is made, and the patient wears this during the day, but has the traction continued for some weeks longer at night. Later on, all cases are applied with a leather spica, which is a combination leather jacket and spica, strengthened at the groin with extra leather and a piece of steel. This may, or may not, include the knee. The case is examined at intervals, for a return of spasm or acute sensitiveness, and ,if they are found, is remanded to bed and traction. The leather spica affords good, although not absolute fixation, and prevents deformity because it keeps the thigh continually in extension in a straight line, without allowing abduction or adduction to any extent. The child is warned to keep from bearing the weight on the heel, and this warning will prevent him from using the leg for the most part, although when sensitiveness has disappeared, all children will disobey to some extent. Massage, and general means, as for tuberculosis elsewhere, are, of course, added. The leather spica is well borne, and a chair, altered at the seat, is provided so that the patient may sit comfortably. The spica is removed at first, only once or twice a week. Later in the course of the disease, it is discarded at night.

It will be seen that this plan provides traction at the same periods, only, that it is used for tubercular joint conditions elsewhere—namely, while there is acute congestion as evidenced by muscular spasm, tenderness, and night cries. In other words, Nature is objecting in her own way, and pointing to a definite pathological state. Lest the large hip muscles, by their contraction, should do harm, the indication is plain that traction is most important. A time will be reached, however, when there is no complaint made if one moves the thigh and gently percusses the heel, knee, trochanter, and structures about the joint. Then fixation, and protection from jar are the major indications. The ultimate aim, in most cases, being to secure anchylosis in a good position. No attempt has been made in this paper to refer to the complications of hip disease, such as abscess or deformity, but the discussion has been confined to the use of traction as applied to the ordinary case.

20 CARLTON STREET.

